# Duration of invasive mechanical ventilation prior to extracorporeal membrane oxygenation is not associated with survival in acute respiratory distress syndrome caused by coronavirus disease 2019

**DOI:** 10.1186/s13613-022-00980-3

**Published:** 2022-01-13

**Authors:** Martina Hermann, Daniel Laxar, Christoph Krall, Christina Hafner, Oliver Herzog, Oliver Kimberger, Sebastian Koenig, Felix Kraft, Mathias Maleczek, Klaus Markstaller, Oliver Robak, Bernhard Rössler, Eva Schaden, Peter Schellongowski, Mathias Schneeweiss-Gleixner, Thomas Staudinger, Roman Ullrich, Marion Wiegele, Harald Willschke, Christian Zauner, Alexander Hermann

**Affiliations:** 1 Department of Anaesthesia, Intensive Care Medicine and Pain Medicine, Medical University of Vienna, Waehringer Guertel 18-20, 1090 Vienna, Austria; 2 Ludwig Boltzmann Institute for Digital Health and Patient Safety, Spitalgasse 23, BT86, 1090 Vienna, Austria; 3 Center for Medical Statistics, Informatics and Intelligent Systems, Medical University of Vienna, Spitalgasse 23, BT88, 1090 Vienna, Austria; 4 Department of Medicine I, Medical University of Vienna, Intensive Care Unit 13i2, Waehringer Guertel 18-20, 1090 Vienna, Austria; 5 Department of Medicine III, Medical University of Vienna, Waehringer Guertel 18-20, 1090 Vienna, Austria

**Keywords:** Acute respiratory distress syndrome, Extracorporeal membrane oxygenation, COVID-19, Invasive mechanical ventilation

## Abstract

**Background:**

Duration of invasive mechanical ventilation (IMV) prior to extracorporeal membrane oxygenation (ECMO) affects outcome in acute respiratory distress syndrome (ARDS). In coronavirus disease 2019 (COVID-19) related ARDS, the role of pre-ECMO IMV duration is unclear. This single-centre, retrospective study included critically ill adults treated with ECMO due to severe COVID-19-related ARDS between 01/2020 and 05/2021. The primary objective was to determine whether duration of IMV prior to ECMO cannulation influenced ICU mortality.

**Results:**

During the study period, 101 patients (mean age 56 [SD ± 10] years; 70 [69%] men; median RESP score 2 [IQR 1–4]) were treated with ECMO for COVID-19. Sixty patients (59%) survived to ICU discharge. Median ICU length of stay was 31 [IQR 20.7–51] days, median ECMO duration was 16.4 [IQR 8.7–27.7] days, and median time from intubation to ECMO start was 7.7 [IQR 3.6–12.5] days. Fifty-three (52%) patients had a pre-ECMO IMV duration of > 7 days. Pre-ECMO IMV duration had no effect on survival (*p* = 0.95). No significant difference in survival was found when patients with a pre-ECMO IMV duration of < 7 days (< 10 days) were compared to ≥ 7 days (≥ 10 days) (*p* = 0.59 and *p* = 1.0).

**Conclusions:**

The role of prolonged pre-ECMO IMV duration as a contraindication for ECMO in patients with COVID-19-related ARDS should be scrutinised. Evaluation for ECMO should be assessed on an individual and patient-centred basis.

**Supplementary Information:**

The online version contains supplementary material available at 10.1186/s13613-022-00980-3.

## Background

Severe coronavirus disease 2019 (COVID-19) predominantly presents with the clinical picture of acute respiratory distress syndrome (ARDS) and a high likelihood of multiple organ failure and death [1]. Venovenous (VV) extracorporeal membrane oxygenation (ECMO) is a known therapeutic option in life-threatening respiratory failure and may improve outcome in ARDS [2]. Although there has been some doubt about the adequacy of ECMO in the context of limited ICU resources, the scientific community has advocated for the use of ECMO in patients with severe COVID-19 ARDS, and notes the crucial role of specialised high-volume centres [3].

During the COVID-19 pandemic, intensive care capacities have repeatedly been exceeded in several regions around the world. From March until May 2020, restrictions placed on the Austrian population prevented the local healthcare system from reaching its capacity limits. However, the resurgence of severe acute respiratory syndrome coronavirus 2 (SARS-CoV-2) infections starting in September 2020 led to the widespread conversion of ordinary ICUs into COVID-19-ICUs to cope with the high influx of COVID-19 patients with respiratory failure. In times of high infection rates, accurate decision-making is vital when it comes to ideal patient allocation. This includes crucial considerations with respect to contraindications and limiting factors, including the duration of invasive mechanical ventilation (IMV) prior to ECMO start. ECMO initiation within 7 days following intubation is considered optimal [4–6] as longer pre-ECMO IMV durations increase mortality in general ARDS populations [7–10]. However, there is no clinically useful cut-off for the maximum antecedent period on IMV.

There is an urgent need for greater understanding of the risk factors influencing mortality in COVID-19 patients receiving ECMO in order to accurately allocate limited ICU and ECMO capacities and avoid triage situations. This retrospective study investigated the effect of pre-ECMO IMV duration on survival and risk factors for dismal outcome.

## Methods

### Study design and setting

Investigator-initiated, retrospective, observational cohort study. This investigation was carried out at the Medical University of Vienna, Austria. In order to meet demand, the Medical University of Vienna converted up to six ICUs into COVID-19 wards designed primarily to provide ECMO support. We included all adult patients treated with ECMO for confirmed COVID-19 in these six ICUs from January 2020 until May 2021. Most of our patients were transferred to our centre from hospitals with no ECMO capability. The observational period ran from ECMO start to ICU discharge at the Medical University of Vienna. This study was approved by the local Ethics Committee of the Medical University of Vienna (EK 2024/2020) and performed in accordance with the Declaration of Helsinki as well as the applicable laws and regulations currently in force. Study design as well as data handling and reporting followed the STROBE guidelines to ensure a maximum level of research quality.

### ECMO management

With respect to the clinical consideration of ECMO, the consultants in charge followed the official Medical University of Vienna consensus recommendations [11]. Details on ECMO evaluation, eligibility assessment, decision-making, implantation technique and management are described ibidem. In accordance with the opinions of international experts [12, 13] and following conventional selection criteria, the use of ECMO in COVID-19-related ARDS was advocated as a last resort option. Thus ECMO was initiated when other strategies including lung protective ventilation, prone positioning, high positive end-expiratory pressure (PEEP), or neuromuscular blocking agents had failed, or in life-threatening hypoxia to avoid cardiopulmonary resuscitation. Our centre adopts a protective ventilation strategy in ARDS patients on ECMO, using a volume-limited controlled ventilation mode pursuing tidal volumes of ≤ 6 ml/kg ideal body weight (IBW), a driving pressure limited to 15 cm H_2_O, and a target peak pressure of ≤ 30 cm H_2_O. We titrate ECMO blood flow to at least 60% of the patient’s cardiac output to maintain peripheral saturation at between 88 and 92%.

### Data sources

Patient identification and data collection were conducted using the patient data management system’s routine documentation (ICCA^©^, Philips, Amsterdam, Netherlands).

The documentation of clinical routine included patient demographic data, underlying disease, reason for hospital/ICU admission, severity of illness on admission expressed by APACHE II score, extent of organ dysfunction expressed by sequential organ failure (SOFA) score, severity of ARDS expressed by respiratory ECMO survival prediction (RESP) score prior to ECMO start, ICU length of stay (LOS), ICU survival, hospital survival, IMV duration prior to ECMO start, and ventilator settings during the course of admission.

Details of ECMO therapy, including duration, and reason for ECMO cessation (e.g., successful weaning, therapy withdrawal, lung transplantation [LTX], death) were extracted. Standard laboratory parameters were routinely documented on a daily basis. Baseline values were collected at the closest timepoint prior to ECMO start, except for one patient whose data were only available from day three onwards.

### Statistical methods

Metric variables were reported using mean and standard deviation (SD) or median and interquartile range (IQR), and ICU survivors compared to non-survivors using t-tests or Mann–Whitney] U tests, according to their distribution, to identify potential risk factors for ICU death. Categorical variables are reported by absolute and relative frequencies, and ICU survivors compared to non-survivors using Chi-squared tests or Fisher’s exact tests, according to their distribution. The primary objective was to determine whether duration of IMV prior to ECMO start influenced ICU mortality. In order to assess the primary objective, a logistic regression model was fitted using ICU mortality as dependent variable, IMV duration prior to ECMO insertion as an independent variable, and age, SOFA score, and RESP score as confounders as these variables showed the greatest differences between survivors and non-survivors in univariate analyses. We used a Chi-squared test to compare the survival of the respective subgroups to address the commonly utilised cut-offs of 7 and 10 days of antecedent IMV duration. In addition, survival analysis was performed to investigate the effects of IMV duration on the hazard. To identify trends in pre-ECMO IMV duration and ICU mortality over time, we analysed a logistic regression model including age, a modified SOFA score (excluding PaO_2_/FiO_2_ ratio), a modified RESP score (excluding age and pre-ECMO IMV duration), and all comorbidities, performing stepwise selection while forcing pre-ECMO IMV to remain in the model. We considered *p* values < 0.05 statistically significant. P values from secondary and exploratory analyses serve only descriptive purposes, hence no multiplicity corrections were applied. Calculations were performed using R statistics software (version 4.0.5, The R Foundation for Statistical Computing, Vienna, Austria).

## Results

### Descriptive data

Between January 2020 and May 2021, we identified 101 consecutive patients admitted to ICU and treated with ECMO for COVID-19-associated ARDS (mean age 56 [SD ± 10] years; 70 [69%] men; median pre-ECMO RESP score 2 [IQR 1–4]; median pre-ECMO SOFA score 8 [IQR 7–10]). Patients had a mean BMI of 31 [SD ± 6] kg/m^2^. Demographic data and detailed information at baseline as well as during ECMO are depicted in Tables 1 and 2, respectively. Median ICU LOS was 31 [IQR 20.7–51] days. The patients’ status over time is visualised by area plot (Additional file 1: Figure S1).

**Table 1:** Demographic data and baseline characteristics, ventilation parameters before ECMO day 0

	All patients *n* = 101	ICU survivors *n* = 60	ICU non-survivors *n* = 41	**p value**
Age, mean (SD)—years	56 (± 10) *n* = 101	52 (± 10) *n* = 60	61 (± 7) *n* = 41	< 0.001
Sex, male, no. (%)	71 (70)	39 (65)	32 (78)	0.2655
BMI, mean (SD)—kg/m^2^	31 (± 6) *n* = 101	31 (± 6) *n* = 60	31 (± 6) *n* = 41	0.946
RESP score, median (IQR)	2 (1–4) *n* = 101	2 (1–4) *n* = 60	1 (0–2) *n* = 41	0.001
SOFA, median (IQR)	8 (7–10) *n* = 101	8 (7–9) *n* = 60	9 (8–11) *n* = 41	0.0002
Apache II, mean (SD)	20 (± 3) *n* = 67	19 (± 3) *n* = 45	21 (± 3) *n* = 22	0.2791
IMV pre-ECMO, median (IQR)—days	7.7 (3.6–12.5) *n* = 101	7.8 (2.5–12.5) *n* = 60	6.8 (4–12.4) *n* = 41	0.9586
IMV < 7 days, no. (%)	48 (48) *n* = 101	27 (45) *n* = 60	21 (51) *n* = 41	0.6805
IMV < 10 days, no. (%)	66 (65) *n* = 101	39 (65) *n* = 60	27 (66) *n* = 41	1
Comorbidities				
Arterial hypertension, no. (%)	60 (59) *n* = 101	32 (53) *n* = 60	28 (68) *n* = 41	0.1946
Coronary artery disease, no. (%)	13 (13) *n* = 101	7 (12) *n* = 60	6 (15) *n* = 41	0.765
Obesity, no. (%)	35 (35) *n* = 101	22 (37) *n* = 60	13 (32) *n* = 41	0.7631
Diabetes mellitus, no. (%)	25 (25) *n* = 101	14 (23) *n* = 60	11 (27) *n* = 41	0.8689
Underlying pulmonary disease, no. (%)	19 (19) *n* = 101	8 (13) *n* = 60	11 (27) *n* = 41	0.1484
Immunosuppression, no. (%)	3 (3) *n* = 101	1 (2) *n* = 60	2 (5) *n* = 41	0.5645
Chronic kidney disease, no. (%)	6 (6) *n* = 101	5 (8) *n* = 60	1 (2) *n* = 41	0.3967
No underlying disease, no. (%)	19 (19) *n* = 101	14 (23) *n* = 60	4 (10) *n* = 41	0.1124
Pre-ECMO				
PaO_2_/FiO_2_, median (IQR)	74.2 (61–109) *n* = 93	82.7 (63.1–121.7) *n* = 54	72.3 (60.3–86.7) *n* = 39	0.1818
PaO_2_, median (IQR)—mmHg	81.8 (70.3–96.4) *n* = 101	82.2 (71.5–94.8) *n* = 60	81 (69.3–96.5) *n* = 41	0.6332
PaCO_2_, median (IQR)—mmHg	47.8 (41.9–56.3) *n* = 101	49.5 (42.6–55) *n* = 60	45.8 (40.9–56.3) *n* = 41	0.2715
pH, median (IQR)—mmHg	7.4 (7.4–7.5) *n* = 101	7.4 (7.4–7.5) *n* = 60	7.4 (7.4–7.5) *n* = 41	0.841
BE, median (IQR)—mmol/L	6.7 (3.9–9.1) *n* = 101	7.1 (4.2–9.3) *n* = 60	5.7 (2.7–8.9) *n* = 41	0.354
Prone positioning, no. (%)	101 (100) *n* = 101	60 (100) *n* = 60	41 (100) *n* = 41	1
iNO, no. (%)	22 (21) *n* = 101	10 (17) *n* = 60	12 (29) *n* = 41	0.4812
Tracheostomy, no. (%)	5 (6)	2 (4)	3 (8)	.
Ventilation pre-ECMO day 0				
PEEP, mean (SD)—cmH_2_O	13 (± 3) *n* = 94	13 (± 4) *n* = 55	13 (± 3) *n* = 39	0.6673
Tidal volume, mean (SD)—ml	443 (± 149) *n* = 94	442 (± 172) *n* = 55	443 (± 110) *n* = 39	0.9854
Tidal volume, mean (SD)—ml/kg IBW	6 (± 2) *n* = 89	6 (± 3) *n* = 54	7 (± 2) *n* = 39	0.7108
Respiratory rate, mean (SD)—/min	22 (± 5) *n* = 94	22 (± 5) *n* = 55	23 (± 5) *n* = 39	0.3307
Plateau pressure, mean (SD)—cmH_2_O	31 (± 5) *n* = 94	31 (± 6) *n* = 55	31 (± 5) *n* = 39	0.5097
Peak pressure, mean (SD)—cmH_2_O	31 (± 5) *n* = 94	31 (± 6) *n* = 55	31 (± 5) *n* = 39	0.5097
Driving pressure, mean (SD)—cmH_2_O	18 (± 5) *n* = 94	18 (± 6) *n* = 55	19 (± 4) *n* = 39	0.3251
Mechanical power, median (IQR)—J/min	30 (22–38) *n* = 94	29 (21–38) *n* = 55	31 (24–37) *n* = 39	0.5142

Metric data are reported by mean (± SD) or by median (IQR), n gives the number of available observations. Variation in RESP score was due almost exclusively to variation in age, therefore we also considered a version of the RESP score without age

*ICU* intensive care unit, *RESP* respiratory *ECMO* survival prediction, *SOFA* Sequential Organ Failure Assessment, *IMV* invasive mechanical ventilation, *BMI* body mass index, *PaO*_*2*_ partial pressure of arterial oxygen, *FiO*_*2*_ fraction of inspired oxygen, *PaCO*_*2*_ partial pressure of arterial carbon dioxide, *BE* base excess, *iNO* inhaled nitric oxide, *IBW* ideal body weight. IBW female = 45.5 + 0.9 * (height [cm]—152); IBW male = 50 + 0.9 * (height [cm]—152); driving pressure = peak pressure—PEEP; mechanical power = 0.098 × respiratory rate × tidal volume (l) × (Δ P_insp_ + PEEP)

**Table 2:** Ventilation parameters on ECMO day 1, ECMO-related data, transfusions, and therapy during clinical course according to ICU mortality

	All patients *n* = 101	ICU survivors *n* = 60	ICU non-survivors *n* = 41	**p value**
ICU LOS, median (IQR)—days	31 (20.7–51) *n* = 100	41 (23.8–58.8) *n* = 60	23 (13.8–36) *n* = 41	0.0011
Lung transplantation, no. (%)	15 (15) *n* = 101	12 (20)	3 (7)	0.0937
Ventilation during ECMO day 1				
PEEP, mean (SD)—cmH_2_O	12 (± 2) *n* = 101	12 (± 2) *n* = 60	13 (± 3) *n* = 41	0.2034
Tidal volume, mean (SD)—ml	270 (± 121) *n* = 101	260 (± 128) *n* = 60	284 (± 112) *n* = 41	0.267
Tidal volume, mean (SD)—ml/kg IBW	4 (± 2) *n* = 101	4 (± 2) *n* = 60	4 (± 2) *n* = 41	0.4715
Respiratory rate, mean (SD)—/min	15 (± 5) *n* = 101	16 (± 6) *n* = 60	15 (± 5) *n* = 41	0.4568
Plateau pressure, mean (SD)—cmH_2_O	26 (± 5) *n* = 101	25 (± 2) *n* = 60	28 (± 5) *n* = 41	0.0076
Peak pressure, mean (SD)—cmH_2_O	26 (± 5) *n* = 101	25 (± 2) *n* = 60	28 (± 5) *n* = 41	0.0076
Driving pressure, mean (SD)—cmH_2_O	14 (± 2) *n* = 101	13 (± 2) *n* = 60	15 (± 4) *n* = 41	0.0191
Mechanical power, median (IQR)—J/min	9 (6.1–13.2) *n* = 101	8.5 (5.5–12.2) *n* = 60	10 (6.5–15.6) *n* = 41	0.2611
ECMO configuration	.	.	.	0.4449
Venoarterial, no. (%)	4 (4)	1 (2)	3 (7)	.
Venovenoarterial, no. (%)	2 (2)	1 (2)	1 (3)	.
Venovenous, no. (%)	95 (95)	58 (97)	38 (93)	.
ECMO duration, median (IQR)—days	16.4 (8.7–27.7) *n* = 101	16.5 (10.8–28) *n* = 60	15 (6.4–24.2) *n* = 41	0.2567
ECMO blood flow, mean (SD)—L/min	3.7 (± 1) *n* = 96	4 (± 1) *n* = 57	4 (± 1) *n* = 39	0.41
During ECMO				
PRBCs, median (IQR)—units per patient	10 (4–15) *n* = 99	9.5 (4.2–15) *n* = 58	11 (4.8–15.5) *n* = 41	0.8449
Albumin, median (IQR)—units per patient	14 (5–27) *n* = 99	13.5 (6–30.8) *n* = 58	15 (5–21.5) *n* = 41	0.4706
Platelet concentrates, no. (%)	20 (20) *n* = 99	9 (16) *n* = 58	11 (27) *n* = 41	0.2599
FFP, no. (%)	16 (16) *n* = 99	12 (21) *n* = 58	4 (10) *n* = 41	0.2386
NMBA, no. (%)	96 (98) *n* = 98	57 (97) *n* = 59	39 (95) *n* = 41	0.5645
Proning, no. (%)	100 (99) *n* = 101	60 (100) *n* = 60	40 (98) *n* = 41	0.4059
iNO, no. (%)	54 (53) *n* = 101	29 (48) *n* = 60	25 (61) *n* = 41	0.3135
Dexamethasone, no. (%)	50 (100) *n* = 101	28 (100) *n* = 60	22 (100) *n* = 41	0.5464
Tracheostomy, no. (%)	44 (51)	32 (67)	12 (32)	.
Outcome				
ICU death, no. (%)	41 (41) *n* = 101	.	.	.
Hospital death, no. (%)	44 (51) *n* = 87	.	.	.

Metric data are reported by mean (± SD) or by median (IQR), n gives the number of available observations

*ICU* intensive care unit, *PEEP* positive end-expiratory pressure, *IBW* ideal body weight, *ECMO* extracorporeal membrane oxygenation, LOS length of stay, *PRBC* packed red blood cells, *FFP* fresh frozen plasma, *NMBA* neuromuscular blocking agents, *iNO* inhaled nitric oxide, *P*_*insp*_ inspiratory pressure. *IBW* female = 45.5 + 0.9 * (height [cm]—152); IBW male = 50 + 0.9 * (height [cm]—152); driving pressure = peak pressure—PEEP; mechanical power = 0.098 × respiratory rate x tidal volume (l) × (Δ P_insp_ + PEEP)

At baseline, mean PEEP was 13 [SD ± 3] cm H_2_O, driving pressure (DP) was 18 [SD ± 5] cm H_2_O, and median mechanical power (MP) was 30 [IQR 22–38] J/min. The last arterial blood gas analysis prior to cannulation showed a median PaO_2_/FiO_2_ ratio of 74.2 [IQR 61–109] mmHg, a median PaCO_2_ of 47.8 [IQR 41.9–56.3] mmHg, and a median pH of 7.4 [IQR 7.4–7.5]. Baseline laboratory values are shown in Additional file 1: Table S1.

### Main findings

By June 2021, 60 patients (59%) were both successfully weaned from ECMO and discharged from ICU. Forty-one patients (41%) died in the ICU. In total 44 (*n* = 87) patients died in-hospital. Three patients died prior to hospital discharge, and 14 patients were lost to follow-up after ICU discharge. Median time from intubation to ECMO start was 7.7 [IQR 3.6–12.5] days in all patients (survivors 7.8 [IQR 2.5–12.5] days, non-survivors 6.8 [IQR 4–12.4] days). Longest period of IMV prior to ECMO was 42 days in a patient who survived after receiving LTX.

Of 53 patients with a pre-ECMO IMV duration > 7 days, 33 patients (62%) survived ICU. Of 35 patients with a pre-ECMO IMV duration > 10 days, 21 patients (60%) survived ICU.

No difference in survival could be identified between patients with ventilation duration of < 7 days (and < 10 days) compared to ≥ 7 days (and ≥ 10 days) using a Chi-squared test (*p* = 0.59 and *p* = 1.0). In addition, the logistic regression for ICU mortality on days of IMV prior to ECMO identified no effect (*p* = 0.95), and the logistic regression for in-hospital mortality on days of IMV prior to ECMO identified no effect (*p* = 0.76), as shown in Table 3. When age, modified SOFA score, modified RESP score, and comorbidities were included in the model to adjust for the baseline condition of the patient, a higher modified SOFA score (*p* = 0.0007), higher modified RESP score (*p* = 0.035), and older age (*p* =  < 0.001) had a positive effect on ICU mortality (Table 4). Even so, no effect on pre-ECMO IMV duration was identified (*p* = 0.199). Underlying pulmonary disease has a marginally positive effect in the presence of the other predictors (*p* = 0.052). Please refer to Additional file 1: Table S2 for the adjusted in-hospital mortality.

**Table 3:** Effect of pre-ECMO IMV duration on mortality in all patients

	Estimate (95% C.I.)	OR (95% C.I.)	*p* value
ICU mortality			
Intercept	−0.367 [−0.971, 0.228]	.	0.2273
Pre-ECMO IMV duration	−0.002 [−0.053, 0.048]	0.998 [0.948, 1.049]	0.9524
Hospital mortality			
Intercept	0.094 [−0.534, 0.728]	.	0.7677
Pre-ECMO IMV duration	−0.008 [−0.06, 0.043]	0.992 [0.941, 1.044]	0.7633

*IMV* invasive mechanical ventilation, *ECMO* extracorporeal membrane oxygenation, *ICU* intensive care unit

**Table 4:** Effect of pre-ECMO IMV duration on ICU mortality in all patients with confounders age, modified SOFA, comorbidities, and modified RESP score

	Estimate (95%C.I.)	OR (95%C.I.)	*p* value
Intercept	−12.141 [−18.812, −6.632]	.	0.0001
Pre-ECMO IMV duration	−0.044 [−0.116, 0.021]	0.957 [0.89, 1.022]	0.1988
Age	0.174 [0.098, 0.267]	1.19 [1.103, 1.022]	< 0.001
SOFA score excluding PaO_2_/FiO_2_	0.403 [0.193, 0.663]	1.496 [1.213, 1.022]	0.0007
RESP score excluding age and IMV	−0.917 [−1.823, −0.099]	0.4 [0.162, 1.022]	0.0346
Underlying pulmonary disease	1.412 [0.033, 2.914]	4.103 [1.033, 1.022]	0.0517

*IMV* invasive mechanical ventilation, *ECMO* extracorporeal membrane oxygenation, *SOFA* Sequential Organ Failure Assessment, *PaO*_*2*_ partial pressure of arterial oxygen, *FiO*_*2*_ fraction of inspired oxygen, *RESP* respiratory ECMO survival prediction, *ICU* intensive care unit

Fifteen patients (15%) received LTX following ongoing ECMO support, of which 12 survived to ICU discharge. No difference in survival was identified between patients with and without LTX (*p* = 0.094). Results of the logistic regression did not change qualitatively when patients with LTX were excluded (Additional file 1: Table S3 and Additional file 1: Table S4).

Baseline characteristics according to pre-ECMO IMV duration are shown in Additional file 1: Table S5 in order to highlight the differences related to IMV duration.

**Fig. 1:**
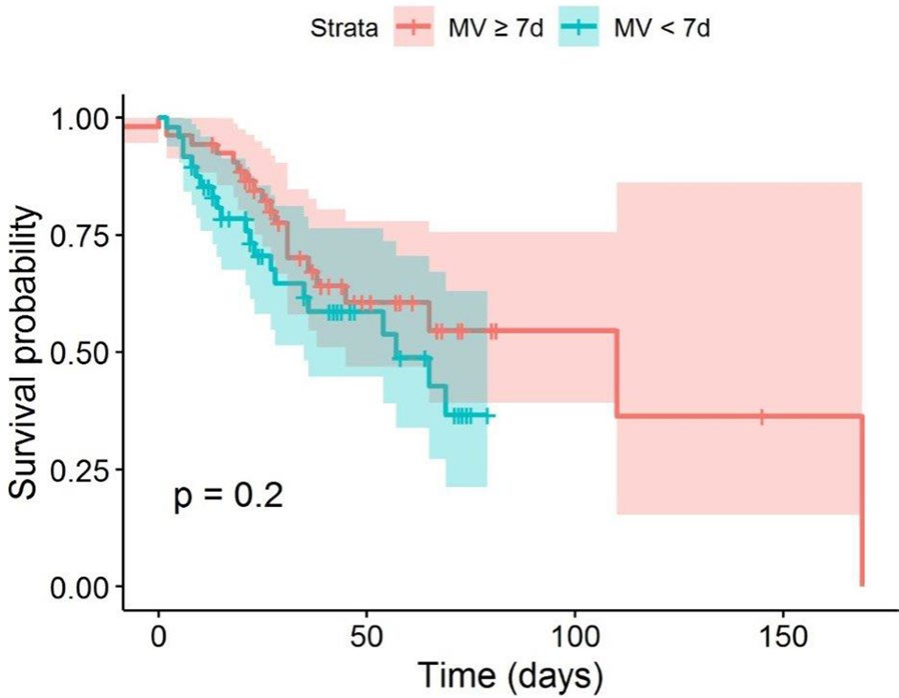
Survival probabilities plotted for all patients grouped by pre-ECMO IMV duration with the cut-off point of 7 days. *ECMO* extracorporeal membrane oxygenation, *IMV* invasive mechanical ventilation

**Fig. 2:**
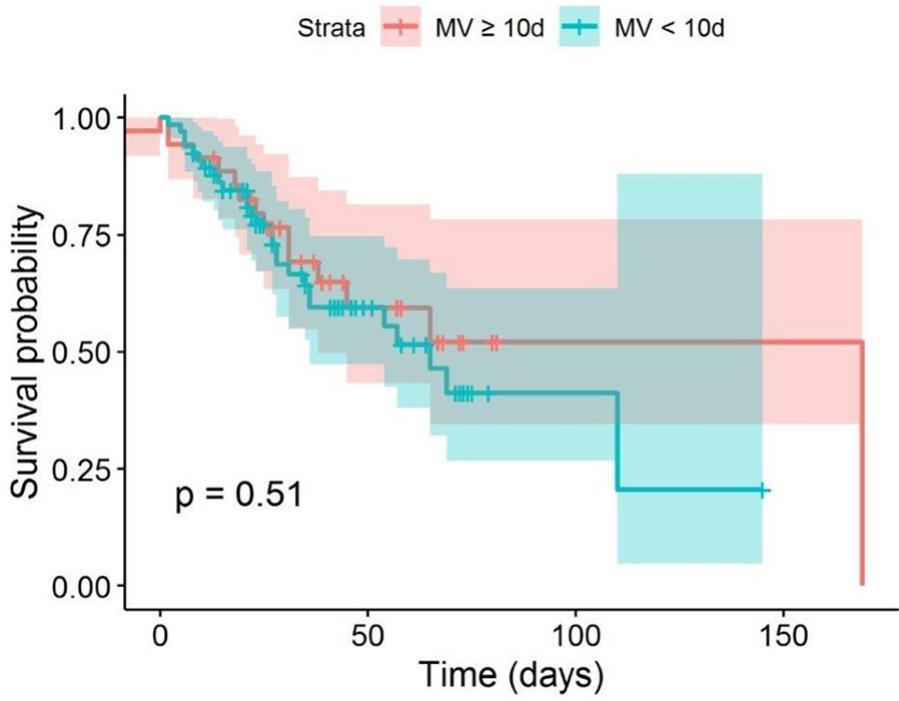
Survival probabilities plotted for all patients grouped by pre-ECMO IMV duration with the cut-off point of 10 days. *ECMO* extracorporeal membrane oxygenation, *IMV* invasive mechanical ventilation

Survival probabilities are plotted for all patients, grouped by pre-ECMO IMV time and with cut-off points of 7 days and 10 days (Figs. 1 and 2). The survival curve for patients with shorter pre-ECMO IMV time is below the curve for patients with longer IMV time for both cut-offs. However, this difference is not significant according to a log-rank test (7 days: *p* = 0.2, 10 days: *p* = 0.51). Survival probabilities for both cut-off points excluding patients with LTX are plotted in Additional file 1: Figure S2 and Additional file 1: Figure S3.

In a Cox regression model including pre-ECMO IMV duration, age, modified SOFA score, modified RESP score, and comorbidities, a reduction of the hazard with longer IMV time was observed (*p* = 0.007, Table 5). Where in-hospital mortality is considered the endpoint, the main qualitative difference to the results for ICU mortality is that the modified RESP score fails to reach significance, possibly due to the smaller sample size with 14 patients lost to follow-up. Again, there was no qualitative change in the results when patients with LTX were excluded (*p* = 0.008, Additional file 1: Table S6).

**Table 5:** Hazard ratios with confidence intervals from a Cox regression model with all patients

	HR (95% C.I.)	*p* value
Pre-ECMO IMV	0.9282 (0.8792, 0.98)	0.0072
Age	1.0909 (0.8792, 1.1438)	0.0003
RESP score excluding age and IMV	0.7798 (0.8792, 1.2859)	0.3298
SOFA score excluding PaO_2_/FiO_2_	1.3092 (0.8792, 1.4904)	< 0.001
Underlying pulmonary disease	1.9651 (0.8792, 4.4373)	0.104

*IMV* invasive mechanical ventilation, *RESP* respiratory ECMO survival prediction, *SOFA* Sequential Related Organ Failure Assessment, *PaO*_*2*_ partial pressure of arterial oxygen, *FiO*_*2*_ fraction of inspired oxygen

**Fig. 3:**
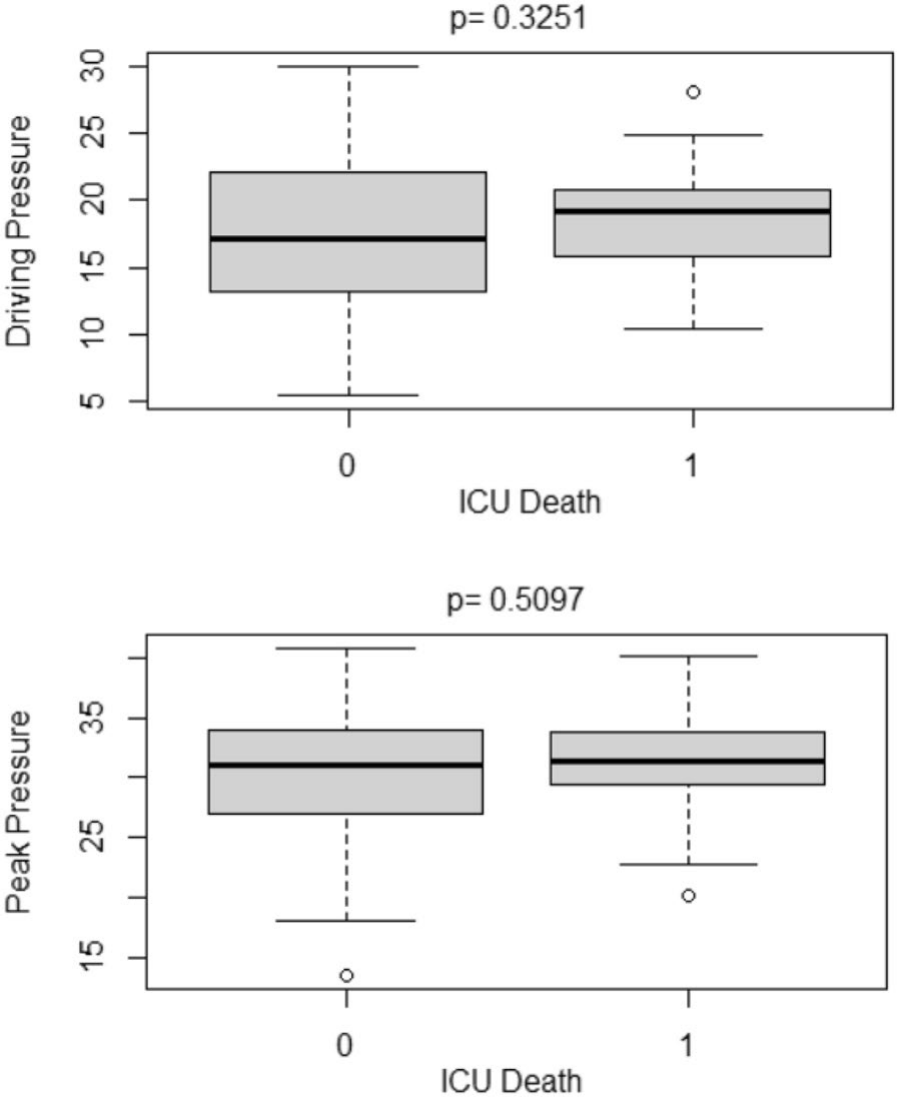
Boxplots of mean DP and mean peak pressure before ECMO according to ICU survival. 0 = ICU survival, 1 = ICU death, *DP* driving pressure, *ICU* intensive care unit

**Fig. 4:**
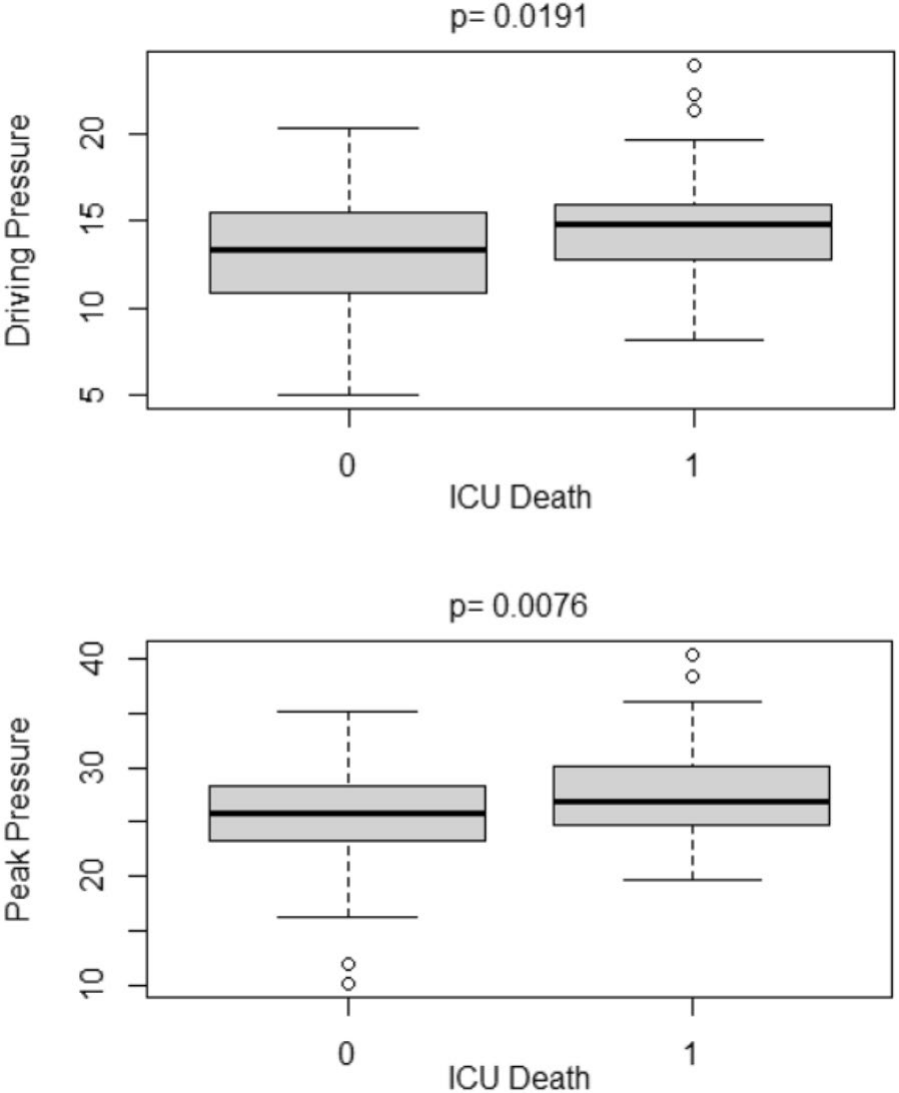
Boxplots of mean DP and mean peak pressure during ECMO according to ICU survival. 0 ICU survival, 1 ICU death, *DP* driving pressure, *ICU* intensive care unit

There was no significant difference in DP and peak pressure prior to ECMO start between survivors and non-survivors (Fig. 3). However, within univariate analyses (*p* = 0.019, *p* = 0.008), both DP and peak pressure in survivors were found to be significantly lower after ECMO start (Fig. 4).

### ECMO-related data

Details of ECMO-related data are shown in Table 2. Indication for VV ECMO was COVID-19-related ARDS in all patients. Indication for venoarterial or venovenoarterial ECMO was COVID-19-related ARDS with associated myocarditis in two patients, extracorporeal cardiopulmonary resuscitation (ECPR) in three patients, and circulatory failure in one patient. All ECPR cases had primarily VV ECMO indications, but hemodynamically deteriorated during cannulation until cardiac arrest occurred. Eight patients (8%) arrived on ECMO, of which five patients were cannulated by the referring hospital and three patients were cannulated in the referring hospital and subsequently retrieved by mobile ECMO teams. All other patients received ECMO in our centre. ECMO-related adverse events are shown in Additional file 1: Table S7.

### Outcome data

Seventy-seven patients (77%) were on ECMO for less than 4 weeks, of which 45 (58%) survived to ICU discharge. Twenty-four patients (23%) were on ECMO for longer than 4 weeks, of which 15 (65%) survived to ICU discharge. This difference was not found significant by a Chi-squared test (*p* = 0.908).

To detect a trend over time, we compared mortality in 2020, when 20 out of 44 patients died (45%), with mortality in 2021, when 21 out of 57 died (36%). Logistic regression showed no significant trend (*p* = 0.79). Mean pre-ECMO IMV duration was 9.7 days in 2020 and 8.5 days in 2021, with no difference in a t-test (*p* = 0.42). A linear regression also identified no correlation (*p* = 0.38).

No effect of pre-ECMO IMV duration on the composed events ICU death or LTX, with confounders age, modified SOFA, and underlying pulmonary disease in particular, could be identified, as shown in Additional file 1: Tables S8 and S9.

The cause of death was multi-organ failure in 30 patients, fatal intracranial haemorrhage in five patients, circulatory failure in five patients, and septic shock in one patient.

## Discussion

In our patient population, the median duration of pre-ECMO IMV was 7.7 days in survivors and 6.8 days in non-survivors. Similar to other observations [14–17], we found no correlation between pre-ECMO IMV duration and survival.

Although some data show the lowest mortality of COVID-19 patients when ECMO initiation takes place within the first three to four days following intubation [18, 19], the current literature provides no clear cut-off for the maximum antecedent time on IMV. In non-COVID-19-associated ARDS, a duration > 7 days has been associated with increased mortality, which is why ECMO initiation, once indicated, should not be delayed [7–9, 20, 21]. Exceeding 7 days from intubation to ECMO is also an integral component in well-established risk prediction scores such as RESP [7] and PRESERVE [8], and has therefore often been considered a relative contraindication for ECMO therapy and thus centre admission [4–6, 22]. During the COVID-19 pandemic, some institutions have considered a limit of 10 days to be more appropriate [23]. Against this background, we compared patients mechanically ventilated for < 7 (and < 10) days with those ventilated for ≥ 7 (and ≥ 10) days in sub-analyses and found no significant difference in ICU survival. It remains uncertain whether ECMO timing in COVID-19 patients should follow commonly utilised entry criteria as stated in the EOLIA trial and our own COVID-19 ECMO guidelines, including long IMV duration as a (relative) contraindication [11, 24]. According to the current norms of practice, prolonged duration of IMV may lead to denial of ECMO therapy, especially in a pandemic context with significant resource constraints [4, 22]. Our findings challenge the applicability of general ECMO entry criteria for COVID-19 patients and the role of pre-ECMO IMV duration of > 7 days as a relative contraindication in commonly utilised recommendations.

In our cohort, ICU survival was 59%. This is similar to previous outcomes for COVID-19 ARDS [5, 14, 15] as well as for severe ARDS resulting from other causes and treated with ECMO [2, 25]. Median duration of IMV before ECMO was 7.7 days, notably exceeding that reported by Barbaro et al. (2.7 to 4 days), Schmidt et al. (4 days), Lebreton et al. (5 days), and Diaz et al. (4 days) [5, 6, 15, 26]. Time of IMV prior to ECMO start was ≥ 7 days in as many as 53 patients and ≥ 10 days in 35 patients, with a maximum of 42 days.

One explanation for the prolonged duration of IMV reported here may be the protracted and often complicated course of severe COVID-19 pneumonia itself [27, 28], although other studies have reported shorter periods on IMV until ECMO was initiated, as mentioned above. However, conservative management had commonly reached its limits in the referring hospitals, potentially leading to late presentation at our institution. Furthermore, longer periods of IMV were regarded as a relative contraindication and therefore tended to be accepted by our consultants where the patient was otherwise eligible for ECMO. This approach could have led to a selection bias, by accepting less sick patients for ECMO treatment. However, pre-ECMO severity of illness expressed by APACHE II showed no differences between survivors and non-survivors. Also, median PaO_2_/FiO_2_ ratio of 74.2 at cannulation expressed profound ARDS severity, similar to other COVID-19 cohorts [2, 14, 25], and did not differ significantly between survivors and non-survivors.

Interestingly, for both cut-offs (7 days and 10 days), the survival probability curve for patients with shorter pre-ECMO IMV duration is below the curve for patients with longer ventilation time, even though this difference is not found to be significant (Figs. 1 and 2). However, this finding is supported by the negative effect of longer IMV on the hazard in survival analysis. In combination with the non-significant effect of IMV duration on ICU mortality, this indicates that pre-ECMO IMV duration does not predict the risk of death in the ICU, but that non-survivors would die sooner if they had shorter IMV durations. In our experience, some patients deteriorate quickly, presenting ECMO indication within a few hours following intubation, while others tolerate IMV for prolonged periods. One explanation for our finding might be that some patients experienced less swift but still aggressive courses of ARDS, leading to slower deterioration and therefore delayed ECMO indication (and thus longer pre-ECMO IMV duration), but still with protracted death. However, this could also happen in the context of variable responses to other treatment cornerstones such as steroid use or prone positioning.

In our patient population, older age was associated with ICU mortality. Age is known to be one of the most important risk factors in COVID-19 patients, with [5, 14] or without ECMO [29]. Usually, patients receiving extracorporeal gas exchange represent a younger population. In our study, however, mean age was 56 years and thus higher than in previously described cohorts [30], reflecting a more liberal attitude to accepting older patients for ECMO implantation. Indeed, our individual decision-making naturally includes an intuitive rating of the biological age, rather than strictly following the chronological age [11]. However, recent observations underline the strong correlation between chronological age and risk of in-hospital mortality [18]. Under pandemic pressure, patient-centred approaches should cautiously consider all aspects known to influence outcome, including both age and health condition, as patients with a high mortality risk commonly require extended ICU resources.

At baseline, RESP score was significantly lower and SOFA score significantly higher in non-survivors. SOFA score is designed to assist prediction of outcome in critically ill patients with organ failure. RESP score has been developed to predict hospital survival at the time of ECMO initiation. Our findings indicate that, even after eliminating components of the RESP score which represent the effects of age and pre-ECMO IMV duration, RESP score is informative for the prediction of ICU mortality in a model including age and pre-ECMO IMV duration separately.

LTX is not a recommended treatment option for COVID-19 ARDS, but may serve as an ultima ratio alternative in highly selected patients with irreversible lung damage [31]. Remarkably, 15 patients in our population received LTX during ECMO therapy, of which 12 patients survived ICU. Indeed, the complexity and risks of post-LTX management may be equal to ongoing ECMO management. The therapy principle of transplantation, however, differs considerably from conventional management, which is why overall comparability may be limited.

For the purposes of generalisation, an additional regression model was fitted using a combined endpoint of either ICU death or lung transplantation (see Additional file 1: Tables S8 and S9) which corresponds to a worst case scenario in which all transplanted patients would have died without LTX. We could not see any effect of IMV on this composed event. Effects of age and SOFA score on prediction of the composed event were comparable to prediction of the event of ICU death alone.

Moreover, when patients receiving LTX were excluded from our statistical analysis, with respect to group differences the results were qualitatively the same throughout, indicating at least no major confounding effect within our population.

One major strength of our study is the high number of patients with longer pre-ECMO IMV durations at our centre compared to previous studies. This accounts for the novel data. Furthermore, our institution does not strictly adhere to predetermined time limits for ECMO support as the decision for therapy cessation is usually based upon individual factors and interdisciplinary discourse.

We acknowledge the following limitations to our study: firstly, the majority of our patients were transferred to our tertiary care centre from a variety of referring hospitals in which IMV had often already been initiated. Detailed information about respiratory management and quality of lung protection is therefore fragmentary. It should be stressed that the strategy and duration of non-invasive ventilation prior to intubation may affect the duration of subsequent IMV, possibly confounding our findings. Secondly, due to our retrospective study design, outcome evaluation allowed for a complete ICU but an incomplete in-hospital survival analysis, the result of missing post-ICU values for 14 patients. It should be emphasised that patients surviving ARDS and ECMO often suffer from relevant post-intensive care sequelae which impair health-related quality of life. In a (post)COVID-19 condition, an even broader range of symptoms may persist which impair daily life and possibly require prolonged hospital stay or rehabilitation [32, 33]. Prospective evaluations with quality-adjusted life years as a patient-centred outcome measure are warranted to depict this highly relevant interval from discharge to recovery. Thirdly, the retrospective nature of our study also accounts for missing data noted in the respective tables. And fourthly, all patients were treated in a high-volume tertiary centre within different departments which may limit the ability to generalise our findings.

## Conclusions

Our data challenge the role of IMV duration as a contraindication for ECMO in severe COVID-19-related ARDS. Although RESP score was a good indicator of survival, an individual approach to balancing both the risks and benefits of ECMO should be pursued. In view of likely further COVID-19 surges, and thereby increased ECMO utilisation, prospective studies are urgently required to determine the optimal practice of ECMO evaluation and its impact on patient-centred outcomes.

## Supplementary Information

**Additional file 1: Figure S1.** Area plot of patient’s status over time. **Figure S2.** Survival probabilities plotted for all patients without LTX, grouped by pre-ECMO IMV duration with the cut-off point of 7 days. LTX = lung transplantation; ECMO = extracorporeal membrane oxygenation; IMV = invasive mechanical ventilation. **Figure S3:** Survival probabilities plotted for all patients without LTX, grouped by pre-ECMO IMV duration with the cut-off point of 10 days. LTX = lung transplantation; ECMO = extracorporeal membrane oxygenation; IMV = invasive mechanical ventilation. **Table S1.** Baseline laboratory values according to ICU mortality. All data are reported by median and IQR and compared using Man–Whitney U tests between groups; n gives the number of available observations. ProBNP = pro-brain natriuretic peptide; ALT = alanine aminotransferase; AST = aspartate aminotransferase; gamma-GT = gamma-glutamyl transferase; LDH = lactate dehydrogenase. **Table S2.** Effect of pre-ECMO IMV duration on hospital mortality in all patients with confounders age, modified SOFA score, comorbidities, and modified RESP score. IMV = invasive mechanical ventilation; ECMO = extracorporeal membrane oxygenation; SOFA = Sequential Organ Failure Assessment; PaO2 = partial pressure of arterial oxygen; FiO2 = fraction of inspired oxygen; RESP = respiratory ECMO survival prediction; ICU = intensive care unit. **Table S3.** Effect of pre-ECMO IMV duration on ICU mortality in all patients after exclusion of patients receiving LTX. IMV = invasive mechanical ventilation; ECMO = extracorporeal membrane oxygenation; ICU = intensive care unit; LTX = lung transplantation. **Table S4.** Effect of pre-ECMO IMV duration on ICU mortality in all patients with confounders age, modified SOFA score and modified RESP score after exclusion of patients receiving LTX. IMV = invasive mechanical ventilation; ECMO = extracorporeal membrane oxygenation; SOFA = Sequential Organ Failure Assessment; RESP = respiratory ECMO survival prediction; ICU = intensive care unit; LTX = lung transplantation. **Table S5.** Baseline characteristics for all patients by pre-ECMO IMV time. Metric data are reported by mean (± SD) or by median and IQR; n gives the number of available observations. IMV = invasive mechanical ventilation; ICU = intensive care unit; LTX = lung transplantation; BMI = body mass index; SOFA = Sequential Organ Failure Assessment. **Table S6.** Hazard ratios with confidence intervals from a Cox regression model after exclusion of patients receiving LTX. IMV = invasive mechanical ventilation; RESP = respiratory ECMO survival prediction; SOFA = Sequential Organ Failure Assessment. **Table S7.** ECMO-related adverse events. All data are reported by numbers and percentages; n gives the number of available observations. GI = gastrointestinal; ECMO = extracorporeal membrane oxygenation. **Table S8.** Effects of pre-ECMO IMV duration on the composed event ICU death or LTX. ICU = intensive care unit; LTX = lung transplantation; IMV = invasive mechanical ventilation; ECMO = extracorporeal membrane oxygenation. **Table S9.** Effect of pre-ECMO IMV duration on the composed event ICU death or LTX in all patients with confounders age, modified SOFA score, and underlying pulmonary disease. IMV = invasive mechanical ventilation; ECMO = extracorporeal membrane oxygenation; SOFA = Sequential Organ Failure Assessment; PaO_2_ = partial pressure of arterial oxygen; FiO_2_ = fraction of inspired oxygen.

## Data Availability

Supporting data from this study can be obtained by emailing the corresponding author.

## Abbreviations

ARDS: Acute respiratory distress syndrome
BMI: Body mass index
COVID-19: Coronavirus disease 2019
DP: Driving pressure
ECMO: Extracorporeal membrane oxygenation
ECPR: Extracorporeal cardiopulmonary resuscitation
IBW: Ideal body weight
ICU: Intensive care unit
IQR: Interquartile range
LOS: Length of stay
LTX: Lung transplantation
MP: Mechanical power
IMV: Invasive mechanical ventilation
PEEP: Positive end-expiratory pressure
RESP score: Respiratory ECMO survival prediction score
SARS-CoV-2: Severe acute respiratory syndrome coronavirus 2
SD: Standard deviation
SOFA: Sequential Organ Failure Assessment
VA: Venoarterial
VV: Venovenous
VVA: Venovenoarterial
