# Supplementary material for: Duration of invasive mechanical ventilation prior to extracorporeal membrane oxygenation is not associated with survival in acute respiratory distress syndrome caused by coronavirus disease 2019
Source: Ann Intensive Care. 2022 Jan 13;12:6. doi: 10.1186/s13613-022-00980-3 (PMC8755897; doi:10.1186/s13613-022-00980-3)
Supplement: Supplementary file 1 — Additional file 1: Figure S1. Area plot of patient’s status over time. Figure S2. Survival probabilities plotted for all patients without LTX, grouped by pre-ECMO IMV duration with the cut-off point of 7 days. LTX = lung transplantation; ECMO = extracorporeal membrane oxygenation; IMV = invasive mechanical ventilation. Figure S3: Survival probabilities plotted for all patients without LTX, grouped by pre-ECMO IMV duration with the cut-off point of 10 days. LTX = lung transplantation; ECMO = extracorporeal membrane oxygenation; IMV = invasive mechanical ventilation. Table S1. Baseline laboratory values according to ICU mortality. All data are reported by median and IQR and compared using Man–Whitney U tests between groups; n gives the number of available observations. ProBNP = pro-brain natriuretic peptide; ALT = alanine aminotransferase; AST = aspartate aminotransferase; gamma-GT = gamma-glutamyl transferase; LDH = lactate dehydrogenase. Table S2. Effect of pre-ECMO IMV duration on hospital mortality in all patients with confounders age, modified SOFA score, comorbidities, and modified RESP score. IMV = invasive mechanical ventilation; ECMO = extracorporeal membrane oxygenation; SOFA = Sequential Organ Failure Assessment; PaO2 = partial pressure of arterial oxygen; FiO2 = fraction of inspired oxygen; RESP = respiratory ECMO survival prediction; ICU = intensive care unit. Table S3. Effect of pre-ECMO IMV duration on ICU mortality in all patients after exclusion of patients receiving LTX. IMV = invasive mechanical ventilation; ECMO = extracorporeal membrane oxygenation; ICU = intensive care unit; LTX = lung transplantation. Table S4. Effect of pre-ECMO IMV duration on ICU mortality in all patients with confounders age, modified SOFA score and modified RESP score after exclusion of patients receiving LTX. IMV = invasive mechanical ventilation; ECMO = extracorporeal membrane oxygenation; SOFA = Sequential Organ Failure Assessment; RESP = respiratory ECMO survival predic [file 13613_2022_980_MOESM1_ESM.docx]

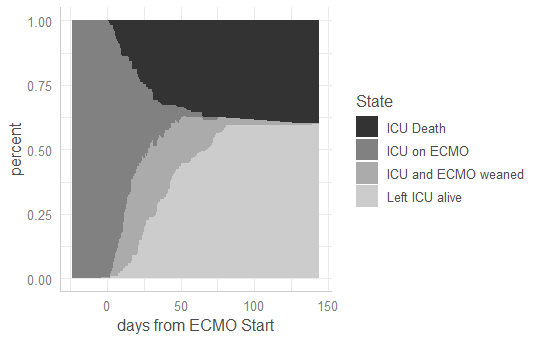


**Additional file 1: Figure S1**


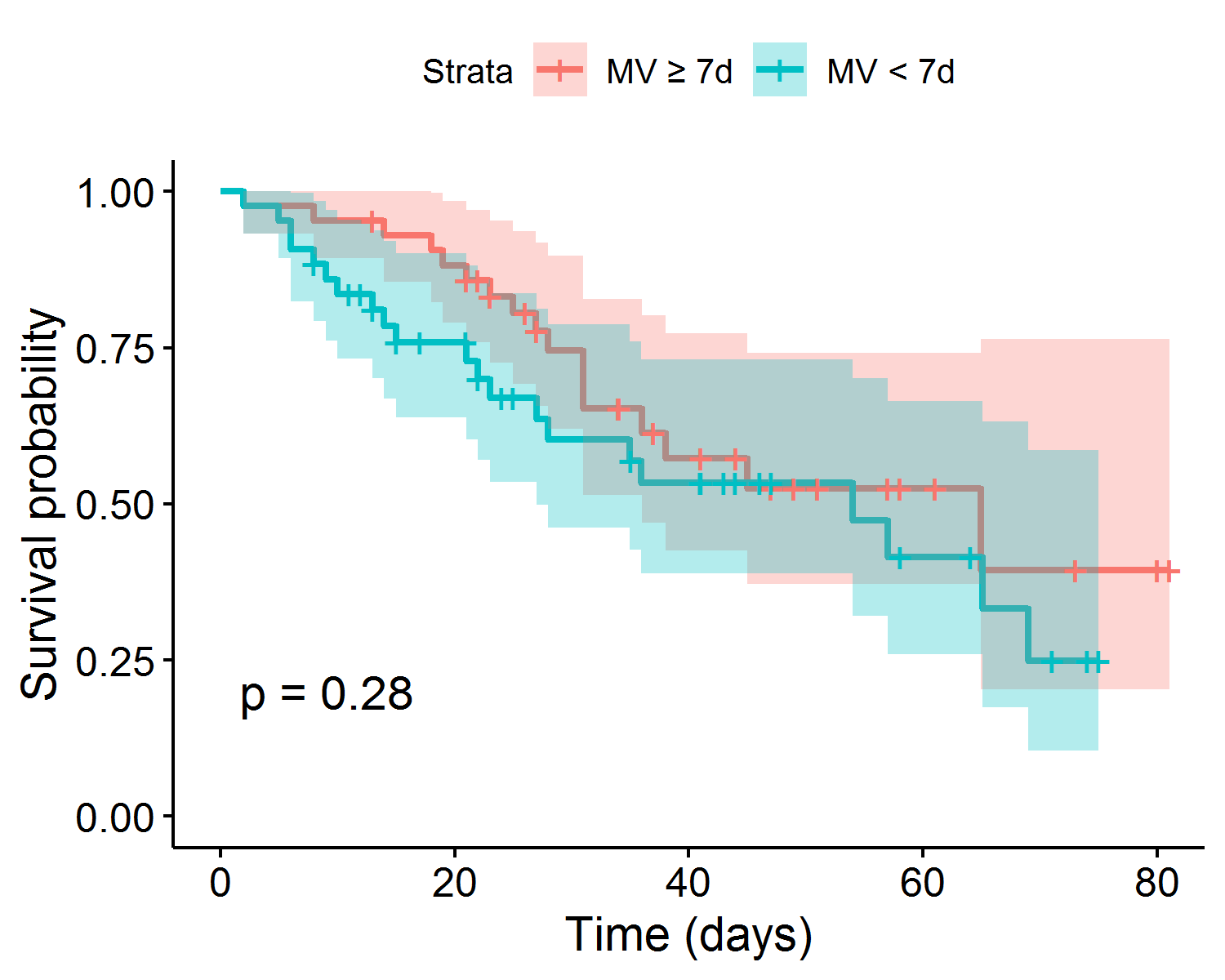


**Additional file 1: Figure S2**


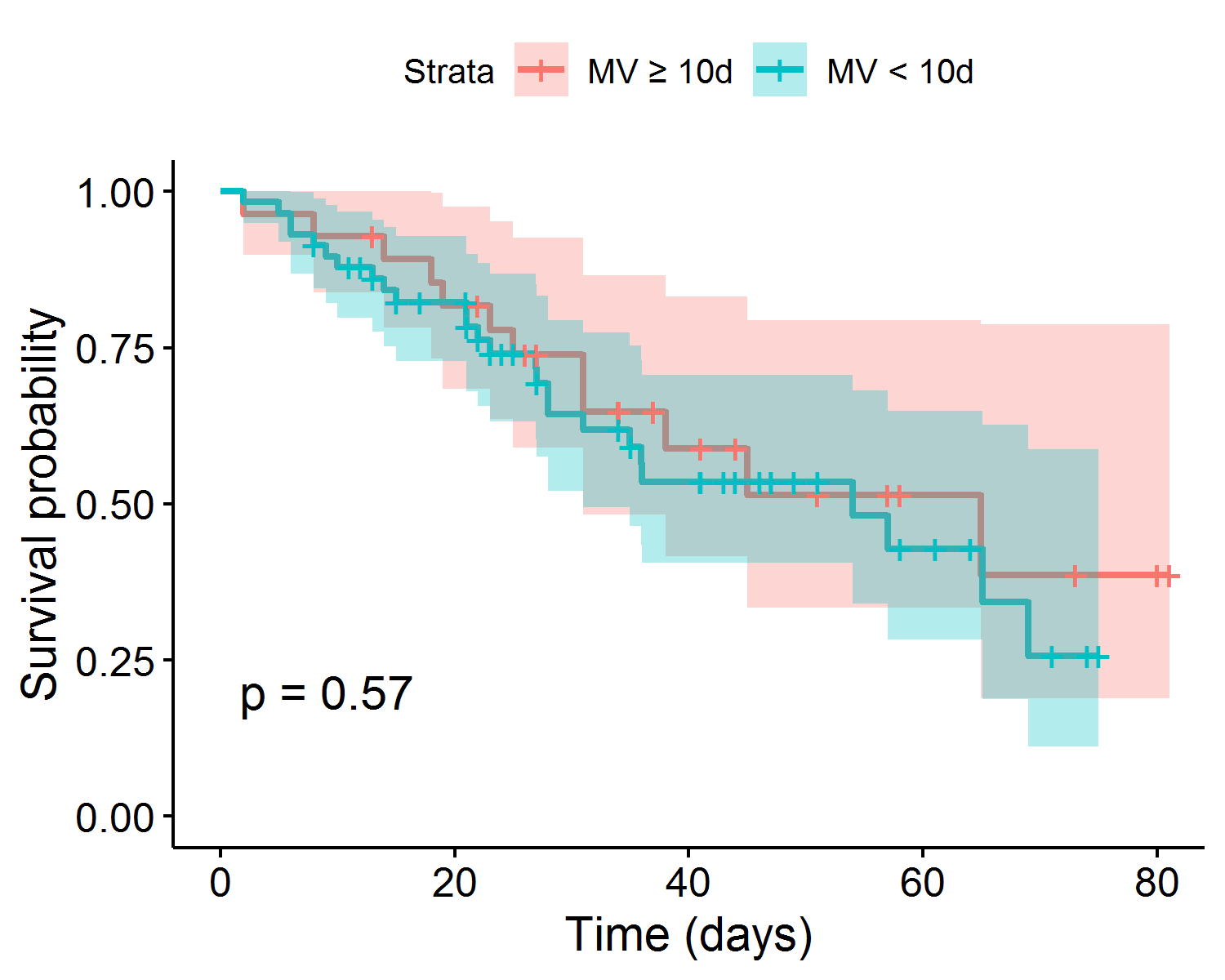


**Additional file 1: Figure S3**

|  | **All patients n=101** | **ICU survivors n=60** | **ICU non-survivors n=41** | **p value** |
| --- | --- | --- | --- | --- |
| Lactate, median (IQR) – mmol/L | 1.5 (1-2) n=101 | 1.2 (0.9-1.8) n=60 | 1.7 (1.3-3) n=41 | 0.0039 |
| Haemoglobin, median (IQR) - g/dl | 9.1 (8.3-10) n=101 | 9.1 (8.3-9.6) n=60 | 9.4 (8.3-10) n=41 | 0.3851 |
| Leukocytes, median (IQR) - /μl | 10.9 (8.5-15.1) n=101 | 10.6 (8-14.6) n=60 | 12.3 (8.9-17.5) n=41 | 0.1417 |
| Platelets, median (IQR) - G/l | 198 (139-240) n=101 | 218 (163-250.2) n=60 | 171 (117-215) n=41 | 0.0248 |
| Creatinine, median (IQR) – mg/dl | 0.7 (0.6-1.2) n=101 | 0.6 (0.4-0.9) n=60 | 1.1 (0.8-1.5) n=41 | <0.001 |
| Urea, median (IQR) – mg/dl | 28.2 (21-40.1) n=101 | 23.8 (19.7-28.8) n=60 | 40 (28.2-50.6) n=41 | <0.001 |
| Sodium, median (IQR) – mmol/L | 144 (142-146) n=101 | 143.5 (141-146) n=60 | 145 (143-146) n=41 | 0.4201 |
| Potassium, median (IQR) – mmol/L | 4.2 (3.9-4.6) n=101 | 4.2 (3.9-4.6) n=60 | 4.3 (4-4.5) n=41 | 0.5396 |
| Calcium, median (IQR) – mmol/L | 2 (1.9-2.1) n=101 | 2 (1.9-2.1) n=60 | 2 (1.9-2.1) n=41 | 0.4301 |
| Magnesium, median (IQR) – mmol/L | 0.9 (0.8-1) n=101 | 0.9 (0.8-1) n=60 | 1 (0.9-1) n=41 | 0.0261 |
| D-Dimer, median (IQR) - μl/ml | 4.3 (2.3-8.2) n=68 | 4.1 (2.6-6) n=39 | 6.7 (2.2-12.5) n=29 | 0.4026 |
| Interleukin-6, median (IQR) – pg/mL | 127(38.2-265.5) n=83 | 81 (29.7-242.5) n=47 | 133 (57.8-320.8) n=36 | 0.2359 |
| Procalcitonin, median (IQR) – ng/mL | 0.7(0.3-1.8) n=84 | 0.4 (0.2-1) n=48 | 1 (0.5-2.1) n=36 | 0.0057 |
| ProBNP, median (IQR) -pg/mL | 688.2 (243.8-2028.5) n=46 | 653.8 (234.7-1446.2) n=26 | 1056.5 (407.2-3266.5) n=20 | 0.3385 |
| Albumin, median (IQR) – g/L | 24 (22.2-28) n=101 | 23.7 (22.2-28.8) n=60 | 24.1 (22.5-27.2) n=41 | 0.9256 |
| AST, median (IQR) -U/L | 50 (35-76.5) n=100 | 47 (30.8-68.2) n=60 | 50 (39.5-92.5) n=40 | 0.1531 |
| ALT, median (IQR) – U/L | 50 (33.8-69.2) n=100 | 52 (35.8-69.2) n=60 | 44 (32-67.8) n=40 | 0.4103 |
| Gamma-GT, median (IQR) -U/L | 166.5 (96.8-316.2) n=100 | 196 (100.5-329.5) n=60 | 140 (79.2-307) n=40 | 0.1532 |
| LDH , median (IQR) – U/L | 395 (324-509) n=100 | 423 (328-511.2) n=60 | 383 (313-486.5) n=40 | 0.5038 |
| Bilirubin total, median (IQR) -mg/dl | 0.8 (0.5-1.6) n=101 | 0.7 (0.5-1.2) n=60 | 1.2 (0.5-2.2) n=41 | 0.042 |

**Additional file 1: Table S1**

|  | **Estimate (95%C.I.)** | **OR (95%C.I.)** | **P value** |
| --- | --- | --- | --- |
| Intercept | -12.94 [-20.669,-6.808] |  | 0.0002 |
| pre ECMO IMV duration | -0.051 [-0.127,0.018] | 0.95 [0.881,1.019] | 0.1573 |
| Age | 0.171 [0.092,0.269] | 1.186 [1.096,1.019] | 0.0001 |
| SOFA score excluding PaO_2_/FiO_2_ | 0.48 [0.226,0.806] | 1.616 [1.254,1.019] | 0.0011 |
| RESP score excluding age and IMV | -0.685 [-1.624,0.182] | 0.504 [0.197,1.019] | 0.1314 |
| Underlying pulmonary disease | 1.55 [0.026,3.259] | 4.711 [1.026,1.019] | 0.0565 |

**Additional file 1: Table S2**

|  | **Estimate (95% C.I.)** | **OR (95% C.I.)** | **p value** |
| --- | --- | --- | --- |
| Intercept | -0.208 [-0.902,0.48] | .. | 0.553 |
| pre ECMO IMV duration | -0.003 [-0.071,0.063] | 0.997 [0.932,1.065] | 0.9249 |

**Additional file 1: Table S3**

|  | **Estimate (95%C.I.)** | **OR (95%C.I.)** | **P value** |
| --- | --- | --- | --- |
| Intercept | -12.141 [-18.812,-6.632] | .. | 0.0001 |
| pre ECMO IMV duration | -0.044 [-0.116,0.021] | 0.957 [0.89,1.022] | 0.1988 |
| Age | 0.174 [0.098,0.267] | 1.19 [1.103,1.022] | <0.001 |
| SOFA score excluding PaO_2_/FiO_2_ | 0.403 [0.193,0.663] | 1.496 [1.213,1.022] | 0.0001 |
| RESP score excluding age and IMV | -0.917 [-1.823,-0.099] | 0.4 [0.162,1.022] | 0.1035 |
| Underlying pulmonary disease | 1.412 [0.033,2.914] | 4.103 [1.033,1.022] | 0.1052 |

**Additional file 1: Table S4**

| **Parameter** | **All patients** | **IMV 7 days +** | **IMV limited 7 days** | **P value** |
| --- | --- | --- | --- | --- |
| Age, mean (SD) – years | 56 (±6), n=101 | 57 (±10), n=53 | 55 (±9), n=48 | 0.4409 |
| Sex, female, no. (%) | 30 (3) | 21 (35) | 9 (22) | 0.235 |
| ICU death, no. (%) | 41 (41) n=101 | 20 (38) n=53 | 21 (44) n=48 | 0.6805 |
| Hospital death, no. (%) | 44 (51) n=87 | 22 (49) n=45 | 22 (52) n=42 | 0.9116 |
| LTX, no. (%) | 15 (15) n=101 | 10 (19) n=53 | 5 (10) n=48 | 0.3615 |
| BMI, mean (SD) – kg/m^2^ | 31 (±6), n=101 | 29 (±6), n=53 | 33 (±6) n=48 | 0.0018 |
| SOFA, median (IQR) | 8 (7-10) n=101 | 8 (7-10) n=53 | 8.5 (7-9.2) n=48 | 0.8335 |
| **COMORBIDITIES** |  |  |  |  |
| Arterial hypertension, no. (%) | 60 (59) n=101 | 30 (57) n=53 | 30 (62) n=48 | 0.6894 |
| Coronary artery disease, no. (%) | 13 (13) n=101 | 6 (11) n=53 | 7 (15%) n=48 | 0.7682 |
| Obesity, no. (%) | 35 (35) n=101 | 10 (19) n=53 | 25 (52) n=48 | 0.001 |
| Diabetes mellitus, no. (%) | 25 (25) n=101 | 15 (28) n=53 | 10 (21) n=48 | 0.5237 |
| Underlying pulmonary disease, no. (%) | 19 (19) n=101 | 8 (15) n=53 | 11 (23) n=48 | 0.4535 |
| Immunosuppression, no. (%) | 3 (3) n=101 | 2 (4) n=53 | 1 (2) n=48 | 1 |
| Chronic kidney disease, no. (%) | 6 (6) n=101 | 4 (8) n=53 | 2 (4) n=48 | 0.6803 |

**Additional file 1: Table S5**

|  | **HR (95% C.I.)** | **P value** |
| --- | --- | --- |
| IMV pre ECMO | 0.9293 (0.8802,0.981) | 0.008 |
| Age | 1.0807 (0.8802,1.1303) | 0.007 |
| RESP score without age and IMV | 0.7408 (0.8802,1.2092) | 0.23 |
| SOFA | 1.3084 (0.8802,1.515) | 0.0003 |
| Chronic respiratory disease | 2.1041 (0.8802,4.7431) | 0.0729 |

**Additional file 1: Table S6**

|  | **All patients n=101** | **ICU Survivors n=60** | **ICU non-survivors n=41** | **p value** |
| --- | --- | --- | --- | --- |
| Unspecific bleeding, no. (%) | 19 (19) n=101 | 12 (20) n=60 | 7 (17) n=41 | 0.9121 |
| Gastrointestinal bleeding, no. (%) | 6 (6) n=101 | 3 (5) n=60 | 3 (7) n=41 | 0.6845 |
| ECMO bleeding, no. (%) | 23 (23) n=101 | 14 (23) n=60 | 9 (22) n=41 | 1 |
| Airway bleeding, no. (%) | 24 (24) n=101 | 12 (20) n=60 | 12 (29) n=41 | 0.4028 |
| Intracranial bleeding, no. (%) | 8 (8) n=101 | 2 (3) n=60 | 6 (15) n=41 | 0.0592 |
| Haemothorax, no. (%) | 7 (7) n=101 | 2 (3) n=60 | 5 (12) n=41 | 0.1166 |
| Pericardial effusion, no. (%) | 6 (6) n=101 | 1 (2) n=60 | 5 (12) n=41 | 0.039 |
| Ischemic stroke, no. (%) | 1 (1) n=101 | 0 (0) n=60 | 1 (2) n=41 | 0.4059 |

**Additional file 1: Table S7**

|  | **Estimate (95%C.I.)** | **OR (95%C.I.)** | **P value** |
| --- | --- | --- | --- |
| Intercept | -0.099 [-0.699,0.491] | .. | 0.7425 |
| pre ECMO IMV duration | 0.022 [-0.027,0.075] | 1.022 [0.974,1.078] | 0.3858 |

**Additional file 1: Table S8**

|  | **Estimate (95%C.I.)** | **OR (95%C.I.)** | **P value** |
| --- | --- | --- | --- |
| Intercept | -9.977 [-15.024,-5.857] | .. | <0.001 |
| pre ECMO IMV duration | 0.016 [-0.045,0.083] | 1.016 [0.956,1.087] | 0.6174 |
| Age | 0.088 [0.035,0.151] | 1.092 [1.036,1.087] | 0.0025 |
| SOFA score excluding PaO_2_/FiO_2_ | 0.413 [0.203,0.669] | 1.511 [1.226,1.087] | 0.0005 |
| Underlying pulmonary disease | 1.127 [-0.099,2.448] | 3.086 [0.906,1.087] | 0.0794 |

**Additional file 1: Table S9**
